# Epidemiology of Human *Mycobacterium bovis* Disease, California, USA, 2003–2011

**DOI:** 10.3201/eid2103.141539

**Published:** 2015-03

**Authors:** Mark Gallivan, Neha Shah, Jennifer Flood

**Affiliations:** California Department of Public Health, Richmond, California, USA (M. Gallivan, N. Shah, J. Flood);; Centers for Disease Control and Prevention, Atlanta, Georgia, USA (N. Shah)

**Keywords:** Tuberculosis, *Mycobacterium bovis*, surveillance, epidemiology, trends, zoonoses, bacteria, California, TB, human infection, genotyping data, tuberculosis and other mycobacteria

## Abstract

We conducted a retrospective review of California tuberculosis (TB) registry and genotyping data to evaluate trends, analyze epidemiologic differences between adult and child case-patients with *Mycobacterium bovis* disease, and identify risk factors for *M. bovis* disease. The percentage of TB cases attributable to *M. bovis* increased from 3.4% (80/2,384) in 2003 to 5.4% (98/1,808) in 2011 (p = 0.002). All (6/6) child case-patients with *M. bovis* disease during 2010–2011 had >1 parent/guardian who was born in Mexico, compared with 38% (22/58) of child case-patients with *M. tuberculosis* disease (p = 0.005). Multivariate analysis of TB case-patients showed Hispanic ethnicity, extrapulmonary disease, diabetes, and immunosuppressive conditions, excluding HIV co-infection, were independently associated with *M. bovis* disease. Prevention efforts should focus on Hispanic binational families and adults with immunosuppressive conditions. Collection of additional risk factors in the national TB surveillance system and expansion of whole-genome sequencing should be considered.

## Abstract 2

Disease was associated with the Hispanic binational population and immunosuppressive conditions, including diabetes.

*Mycobacterium bovis*, part of the *Mycobacterium tuberculosis* complex, is a zoonotic pathogen that can cause tuberculosis (TB) disease in a broad range of mammalian hosts (*1*). TB disease caused by *M. bovis* is clinically, radiographically, and pathologically indistinguishable from TB caused by *M. tuberculosis* (*2*). *M. bovis* transmission to humans most frequently occurs through consumption of unpasteurized, contaminated dairy products, but person-to-person transmission has been reported (*3*,*4*). The consumption of contaminated unpasteurized dairy products has been suggested as a major contributor to human *M. bovis* disease for several reasons: 1) the near absence of *M. bovis* disease among infants <12 months of age; 2) a high percentage of extrapulmonary disease, particularly abdominal disease, among patients with *M. bovis* disease; and 3) an association between positive interferon-γ release assay results and consumption of unpasteurized dairy products (*5*–*11*).

In the United States, 1%–2% of all human TB cases are attributable to *M. bovis* infection (*7*), but in certain geographic regions and communities, human *M. bovis* infection accounts for a much higher percentage of the cases. During 2001–2005, *M. bovis* accounted for nearly 10% of culture-positive TB isolates in San Diego, California, USA, including 54% of those from children (<15 years of age) and 8% of those from adults (>15 years of age) (*8*). Nearly all (97%) case-patients with *M. bovis* disease were among the Hispanic population, and 60% of those case-patients were born in Mexico (*8*). During 2001–2004, an investigation in New York, New York, USA, showed a high prevalence of TB caused by *M. bovis* among the Hispanic community. New York investigators reported that 57% of *M. bovis* case-patients were born in Mexico, and 83% of the interviewed case-patients consumed unpasteurized cheeses produced in Mexico while living in the United States (*12*).

The internationally recognized genotypic method for identifying *M. bovis* is spacer oligonucleotide typing (*13*). In 2004, the CDC (Centers for Disease Control and Prevention) Tuberculosis Genotyping Program (now called the National Tuberculosis Genotyping Service, http://www.cdc.gov/tb/publications/factsheets/statistics/genotyping.htm) began spoligotyping *M. tuberculosis* complex isolates from US patients with culture-positive TB (*14*). In California, the percentage of culture-positive isolates spoligotyped each year has gradually increased from 35% in 2004 to 92% in 2011. The incompleteness and variability (by geographic location) of spoligotype testing over this period exclude trend analysis and population-based *M. bovis* studies using this genotypic method. However, pyrazinamide monoresistance can serve as a proxy measure for *M. bovis* because *M. bovis* is intrinsically resistant to pyrazinamide but pyrazinamide monoresistance is rare among *M. tuberculosis* isolates (*7*,*15*,*16*). A recent national study on pyrazinamide resistance showed that 0.7% (196/27,428) *M. tuberculosis* isolates were pyrazinamide monoresistant (*15*). Since 2003, ≈97% of all culture-positive TB isolates in California have had initial (i.e., pretreatment) drug susceptibility testing for pyrazinamide, isoniazid, and rifampin.

*M. bovis* disease is of particular concern because of the high percentage of cases among children and because of its association with zoonotic and foodborne transmission, HIV co-infection, and poor treatment outcomes compared with *M. tuberculosis* disease (*6*,*17*–*19*). Further investigation into *M. bovis* disease is needed to understand the epidemiology of cases among children and adults, its association with immunosuppressive conditions, and the association of those conditions with treatment outcomes. We conducted a retrospective review of California TB surveillance data to evaluate trends for TB cases attributable to *M. bovis*, evaluate epidemiologic differences between *M. bovis* TB cases in adults and children, and identify risk factors associated with *M. bovis* disease compared with *M. tuberculosis* disease. We also conducted an evaluation of the accuracy of pyrazinamide monoresistance as a proxy measure for *M. bovis* disease by using surveillance and genotyping data.

## Methods

The study population included all patients with culture-confirmed TB reported to the California TB registry during 2003–2011 and who had initial drug susceptibility testing results for isoniazid, rifampin, and pyrazinamide. Patients were classified as having *M. bovis* disease if the initial drug susceptibility results indicated resistance to pyrazinamide and susceptibility to isoniazid and rifampin. Sociodemographic, clinical, and treatment outcome information for all case-patients was abstracted from the TB registry. TB case registry data were matched to the California HIV/AIDS registry to identify HIV co-infection status. This analysis was conducted as part of the California Department of Public Health’s mandate to routinely collect and analyze surveillance data for public health purposes. The CDC (Atlanta, GA, USA) determined that the project was not human subject research and did not require approval by an institutional review board.

To compare the differences between child and adult populations, we stratified *M. bovis* case-patients by their age at the time TB was reported. The child population consisted of patients <15 years of age, and the adult population consisted of patients >15 years of age. To evaluate characteristics associated with TB disease caused by *M. bovis* compared with TB disease caused by *M. tuberculosis*, we conducted bivariate analysis with variables that were added to the national TB surveillance system in 2010. These variables include birth country of parents/guardians (for child case-patients), primary reason evaluated for TB disease, diabetes mellitus, and other immunosuppressive conditions. Immunosuppressive conditions, excluding HIV co-infection and diabetes mellitus, comprised end-stage renal disease, anti–tumor necrosis factor-α therapy–associated immunosuppression, solid organ transplant–associated immunosuppression, or other immunosuppressive condition as indicated by medical records or a health care provider. In addition, patient variables previously shown to be associated with TB disease caused by *M. bovis* (i.e., country of birth, race/ethnicity, age, HIV co-infection, site of disease, and death before treatment completion) were included in the bivariate analysis (*7*,*19*). Sociodemographic and clinical variables shown to be significantly associated with *M. bovis* disease at the bivariate level were put into a logistic regression model. The final logistic regression model was constructed by using the backward stepwise elimination procedure, removing predictors with p>0.05.

For TB cases during 2004–2011, the sensitivity and positive predictive value of the pyrazinamide monoresistance case definition were calculated by using spoligotyping data as the reference standard. The signature spoligotypes differentiating *M. bovis* and *M. tuberculosis* have been described previously in detail (*20*). Spoligotyping was conducted at the California Microbial Diseases Laboratory (Richmond, CA, USA), and the resulting data were entered into the TB Genotyping Information Management System (http://www.cdc.gov/tb/programs/genotyping/tbgims/default.htm). The TB registry and TB Genotyping Information Management System databases were merged by patients’ unique case numbers after spoligotyping data were deduplicated. Spoligotyping results indicating a genotype other than *M. bovis* or *M. tuberculosis* (including *M. bovis* bacillus Calmette-Guérin (BCG) strains) and isolates missing spoligotyping data or initial drug susceptibility testing results for pyrazinamide, isoniazid, or rifampin were excluded from analysis

Analyses were conducted by using SAS version 9.3 (SAS Institute, Cary, NC, USA). Separate Poisson regression models were used to identify temporal trends in the incidence of TB caused by *M. tuberculosis* and *M. bovis*. Year of TB case report was used as the explanatory variable, TB case number as the dependent variable, and state population size as the offset variable. Population denominators were obtained from the US Census Bureau’s current population survey (*21*). Trends of the annual percentage of TB cases attributable to *M.* bovis were examined by using the Cochran-Armitage trend test. Epidemiologic differences between *M. bovis* and *M. tuberculosis* were compared by using the χ^2^ test or Fisher exact test. Differences in median time to completion of therapy from start of therapy were analyzed by using the Wilcoxon rank-sum test. Trends and differences in disease characteristics were considered statistically significant if p<0.05 (2-sided).

## Results

### Burden and Trends

**Figure 1:**
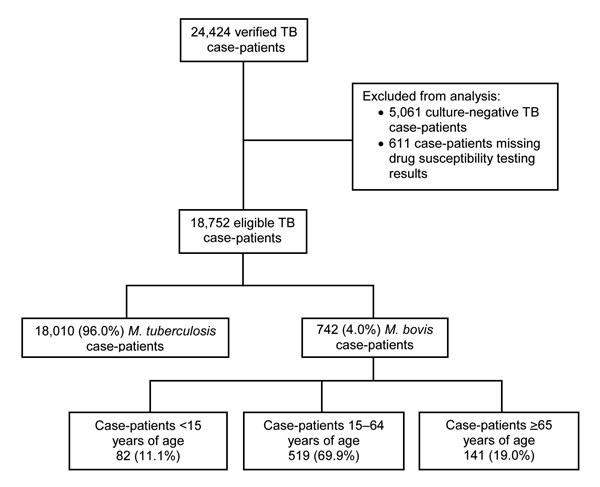
Number of verified tuberculosis case-patients eligible for inclusion in an epidemiologic study of human *Mycobacterium bovis* disease, California, USA, 2003–2011.

**Figure 2:**
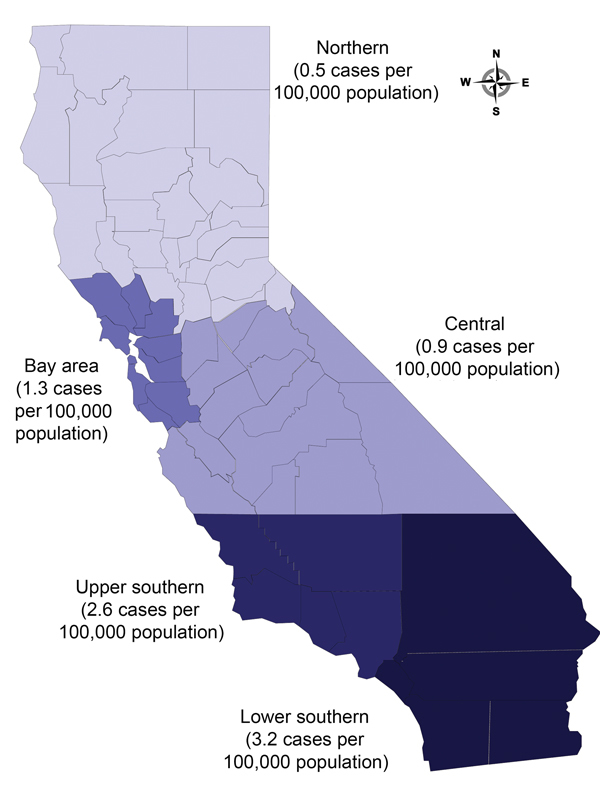
Cumulative incidence of human *Mycobacterium bovis* disease, by region, California, USA, 2003–2011.

During 2003–2011, a total of 24,424 verified TB cases were reported in California. Of these, 5,061 (21.0%) culture-negative cases were excluded (Figure 1). Approximately 3.0% (611/19,363) of culture-positive cases were excluded because results of initial drug susceptibility testing for isoniazid, rifampin, or pyrazinamide were absent. *M. bovis* was identified by pyrazinamide monoresistance in 4.0% (742/18,752) of all eligible TB cases. *M. bovis* accounted for 22.0% (82/379) of culture-positive TB cases in children, 3.9% (519/13,397) of cases in adults 15–64 years of age, and 2.8% (141/4,976) of cases in adults >65 years of age. A total of 163 children <12 months of age had TB disease, and among those with culture-positive cases (81 children), none had *M. bovis* disease. The highest cumulative incidence of human *M. bovis* disease occurred in lower southern California (Figure 2).

**Figure 3:**
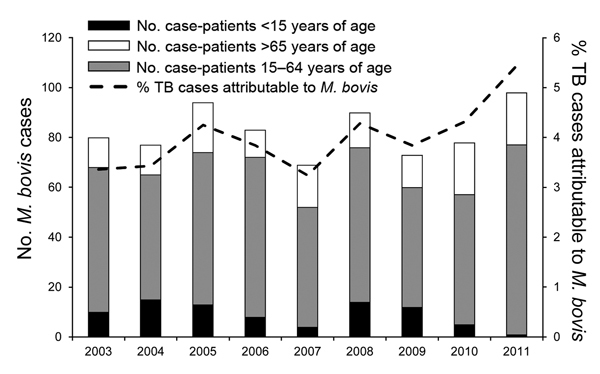
Annual number of case-patients with *Mycobacterium bovis* disease and percentage of tuberculosis cases attributable to *M. bovis*, California, USA, 2003–2011.

During 2003–2011, TB incidence attributable to *M. tuberculosis* declined significantly (p<0.0001), but TB incidence attributable to *M. bovis* did not change (p = 0.92). The annual percentage of TB cases attributable to *M. bovis* among all age groups increased from 3.4% (80/2,384) in 2003 to 5.4% (98/1,808) in 2011 (p = 0.002; Figure 3). The annual percentage of TB cases attributable to *M. bovis* among the child population did not change significantly (p = 0.15), but the percentage among adults increased from 3.0% to 5.5% (p<0.001).

### Epidemiology of *M. bovis* Disease in Children versus Adults

Several differences were observed between child and adult case-patients with *M. bovis* disease (Table 1). Child case-patients were more likely to be Hispanic and US-born and to have extrapulmonary disease. All *M. bovis* case-patients with concurrent HIV co-infection were adults. During 2003–2011, a total of 11.4% (80/699) of all case-patients with *M. bovis* disease died before treatment was completed. Among children, 1.2% (1/82) died before treatment was completed, compared with 12.8% (79/617) of adults (p = 0.002). Among case-patients who completed treatment, the median time to completion did not differ significantly between children and adults.

**Table 1:** Sociodemographic and clinical characteristics of and treatment outcomes for case-patients with tuberculosis attributable to *Mycobacterium bovis*, California, USA, 2003–2011*

Characteristic or outcome	No. (%) patients, by age group		p value	No. patients/no. total (%), N = 742†
	<15 y, n = 82	>15 y, n = 660		
Sex			0.08	
F	47 (57.3)	311 (47.1)		
M	35 (42.7)	349 (52.9)		384 (52)
Race/ethnicity			0.0007	
Hispanic	81 (98.8)	518/659 (78.6)		599/741 (80.7)
Asian, non-Hispanic	1 (1.2)	99/659 (15.0)		100/741 (13.5)
Black, non-Hispanic	0	8/659 (1.2)		8/741 (1.1)
White, non-Hispanic	0	29/659 (4.4)		29/741 (3.9)
Other	0	5/659 (0.8)		5/741 (0.7)
Country of birth			<0.0001	
United States	70 (85.4)	112 (17.0)		182 (24.5)
Mexico	11 (13.4)	426 (64.5)		437 (58.9)
Other†	1 (1.2)	122 (18.5)		123 (16.6)
Disease site			<0.0001	
Extrapulmonary only	71 (86.6)	248 (37.6)		319 (43.0)
Pulmonary only	3 (1.1)	266 (40.3)		269 (36.3)
Extrapulmonary and pulmonary	8 (5.2)	146 (22.1)		154 (20.7)
Positive acid-fast bacilli sputum smear‡	2/7 (28.6)	223/390 (57.2)	0.25	225/397 (56.7)
Presence of cavitation on chest radiographs‡	1/8 (12.5)	89/383 (23.2)	0.69	90/391 (23.0)
Reported HIV co-infection	0 (0.0)	83 (12.6)	<0.0001	83 (11.2)
Health care provider type			0.002	
Health department	16 (19.5)	252/644 (39.2)		268/726 (37.0)
Private/other	43 (52.4)	271/644 (42.0)		314/726 (43.2)
Health department and private/other	23 (28.1)	121/644 (18.8)		144/726 (19.8)
Dead at diagnosis	0 (0.0)	20 (3.5)	0.15	20 (3.1)
Treatment outcome			0.002	
Patient died before completion	1/82 (1.2)	79/617 (12.8)		80/699 (11.4)
Completed treatment	79/82 (96.4)	511/617 (82.8)		590/699 (84.4)
Patient lost during treatment	1/82 (1.2)	12/617 (2.0)		13/699 (1.9)
Refused treatment	1/82 (1.2)	2/617 (0.3)		3/699 (0.4)
Other	0/82 (0.0)	13/617 (2.1)		13/699 (1.9)
Median no. months to treatment completion (IQR)§	10.1 (9.1, 12.4)	9.4 (8.9, 12.0)	0.16	9.5 (8.9, 12.0)

*Data are no. (%) case-patients, except as noted. n values are indicated for categories with missing or incomplete data. AFB, acid-fast bacilli; IQR, interquartile range. †Other countries (no. cases): Vietnam (33), Philippines (18), El Salvador (13), Cambodia (11), China (9), India (7), Indonesia (4), Guatemala (3), Peru (3), North Korea (3), Fiji (2), South Korea (2), Thailand (2), Cameroon (1), Chile (1), Egypt (1), Ethiopia (1), Honduras (1), Iran (1), Japan (1), Laos (1), Malaysia (1), Nicaragua (1), Taiwan (1), Tanzania (1), and Ukraine (1). ‡Among tested patients with any pulmonary disease involvement. §Among patients who completed therapy.

### *M. bovis* Epidemiology versus *M. tuberculosis* Epidemiology

Bivariate analysis showed that the following patient characteristics were associated with TB caused by *M. bovis* but not by TB caused by *M. tuberculosis*: Hispanic ethnicity, birth in Mexico, incidental laboratory result as primary reason for TB evaluation, HIV co-infection, presence of extrapulmonary TB disease, and diabetes and other immunosuppressive conditions. Results were similar when we repeated the bivariate analysis using the spoligotyping method to identify *M. bovis* disease. A higher percentage of *M. bovis*– than *M. tuberculosis*–infected patients had diabetes; however, this association was no longer significant (p = 0.08) because the study population was smaller. The association between *M. bovis* disease and other immunosuppressive conditions remained significant (p = 0.002).

During 2010–2011, case-patients with *M. bovis* disease were more likely than those with *M. tuberculosis* disease to die before treatment completion (15.8% vs. 8.6%, p = 0.006) (Table 2). Among case-patients who died before treatment completion, those with *M. bovis* disease were more likely than those with *M. tuberculosis* disease to have had >1 concurrent immunosuppressive condition (73% vs. 53%, p = 0.05). Of the 26 *M. bovis* case-patients who died before treatment completion, 19 (73%) had >1 concurrent immunosuppressive condition: diabetes and end-stage renal disease (n = 6), diabetes (n = 5), HIV co-infection (n = 1), end-stage renal disease (n = 1), post–organ transplantation–associated immunosuppression (n = 1), end-stage renal disease and post-organ transplantation–associated immunosuppression (n = 1), or any other immunosuppressive condition (n = 4).

**Table 2:** Sociodemographic and clinical characteristics of and treatment outcomes for case-patients with tuberculosis attributable to *Mycobacterium bovis* and *M. tuberculosis*, California, USA, 2003–2011

Patient group, characteristic or outcome	No. (%) cases*		p value
	*M. bovis*, n = 176	*M. tuberculosis,* n = 3,445	
Child case-patients†			
Country of birth			0.02
United States	4/6 (66.7)	54/58 (93.1)	
Mexico	2/6 (33.3)	1/58 (1.7)	
Other‡	0/6	3/58 (5.2)	
Any parent/guardian born in Mexico	6/6 (100.0)	22/58 (38.0)	0.005
Lived in Mexico for >2 mo	4/6 (66.7)	2/58 (3.4)	<0.001
All patients			
Age, years			0.24
0 to <15	6 (3.4)	58 (1.7)	
15–24	16 (9.1)	304 (8.8)	
25–44	46 (26.1)	990 (28.7)	
45–64	66 (37.5)	1,112 (32.3)	
>65	42 (23.9)	981 (28.5)	
Race/ethnicity			<0.001
White, non-Hispanic	3 (1.7)	277 (8.0)	
Black, non-Hispanic	3 (1.7)	204 (5.9)	
Asian, non-Hispanic	42 (23.9)	1,743 (50.6)	
Hispanic	127 (72.2)	1,187 (34.5)	
Other	1 (0.6)	34 (1.0)	
Country of birth			<0.001
United States	33/175 (18.9)	643/3,438 (18.7)	
Mexico	94/175 (53.7)	740/3,438 (21.5)	
Other country‡	48/175 (27.4)	2,057/3,438 (59.8)	
Main site of disease			<0.001
Pulmonary	110 (62.5)	2,938 (85.2)	
Extrapulmonary only	66 (37.5)	507 (14.8)	
Presence of cavitation on chest radiographs§	23/99 (23.2)	666/2,781 (24.0	0.87
Positive acid-fast bacilli sputum smear§	57/101 (56.4)	1,678/2,785 (60.3)	0.44
Reported HIV co-infection	13 (7.4)	140 (4.1)	0.03
Presence of diabetes mellitus	59 (33.5)	826 (24.0)	0.004
Presence of other immunosuppressive condition(s)¶	35 (19.9)	352 (10.2)	<0.001
Primary reason evaluated for tuberculosis			<0.001
Presence of tuberculosis symptoms	116 (65.9)	2,243/3,435 (65.3)	
Abnormal results on chest radiograph	26 (14.8)	608/3,435 (17.7)	
Contact investigation	0	86/3,435 (2.5)	
Targeted testing	2 (1.1)	60/3,435 (1.7)	
Immigration medical examination	3 (1.7)	113/3,435 (3.3)	
Incidental laboratory result	29 (16.5)	285/3,435 (8.3)	
Other	0	40/3,435 (1.2)	
Treatment outcome			0.006
Patient died before completion	26/165 (15.8)	285/3,299 (8.6)	
Completed treatment	126/165 (76.3)	2,786/3,299 (84.4)	
Other	13/165 (7.9)	228/3,299 (6.9)	

*n values are indicated for categories with missing or incomplete data. †Case-patients <15 y of age.  ‡Other countries (no. cases): Ethiopia (1), Malaysia (1), Vietnam (1). §Of case-patients with any pulmonary involvement. ¶Includes end-stage renal disease, immunosuppression associated with receiving anti–tumor necrosis factor-α therapy, immunosuppression associated with receiving solid-organ transplant, or other immunosuppressive conditions, but excludes diabetes and HIV co-infection.

Subanalysis of child case-patients during 2010–2011 showed that 100% (6/6) of children with *M. bovis* disease had >1 parent/guardian who had been born in Mexico, compared with 38% (22/58) of children with *M. tuberculosis* disease (p = 0.005). In addition, 66% (4/6) of the children with *M. bovis* disease were born in the United States and 33% (2/6) were born in Mexico, compared with 93.1% (54/58) and 1.7% (1/58), respectively, of the children with *M. tuberculosis* disease (p = 0.02). Moreover, 66% (4/6) of the children with *M. bovis* disease had lived in Mexico for >2 months, compared with 3% (2/58) of the children with *M. tuberculosis* disease (p<0.001).

Multivariate analysis showed that Hispanic ethnicity (compared with non-Hispanic white), extrapulmonary disease, and diabetes and other immunosuppressive conditions were independently associated with *M. bovis* disease compared with *M. tuberculosis* disease (Table 3). Birth in a country other than the United States, excluding Mexico, was negatively associated with *M. bovis* disease compared with *M. tuberculosis* disease. Birth in Mexico was not independently associated with *M. bovis* disease (adjusted odds ratio 1.1, 95% CI 0.7–1.8).

**Table 3:** Multivariate model of sociodemographic and clinical characteristics associated with *Mycobacterium bovis* disease versus *M. tuberculosis* disease, California, USA, 2010–2011

Risk factors	Adjusted odds ratio (95% CI)
Diabetes status	
Absent	Reference
Present	1.5 (1.1–2.1)
Other immunosuppressive condition(s)*	
Absent	Reference
Present	1.8 (1.2–2.8)
Race/ethnicity	
White, non-Hispanic	Reference
Black, non-Hispanic	1.2 (0.2–6.2)
Asian, non-Hispanic	3.6 (1.0–12.8)
Hispanic	7.3 (2.2–24.3)
Other	2.3 (0.2–23.3)
Country of birth	
United States	Reference
Mexico	1.1 (0.7–1.8)
Other	0.4 (0.2–0.7)
Main site of disease	
Pulmonary†	Reference
Extrapulmonary only	3.9 (2.8–5.5)

*Excludes diabetes mellitus and HIV co-infection but includes end-stage renal disease, immunosuppression associated with receiving anti–tumor necrosis factor-α therapy, immunosuppression associated with receiving solid-organ transplant, or other immunosuppressive condition. †Includes case-patients with pulmonary and extrapulmonary disease.

### Evaluation of the Pyrazinamide Monoresistance *M. bovis* Definition

Among all isolates with spoligotype and drug resistance data available during 2004–2011, the pyrazinamide monoresistance case definition had a sensitivity of 92% (95% CI 90%–95%) and positive predictive value of 82% (95% CI 79%–86%) for *M. bovis*. Among the Hispanic population, the sensitivity and positive predictive value of the pyrazinamide monoresistance definition was 94% (95% CI 92%–97%) and 96% (95% CI 94%–98%), respectively. When the analysis was restricted to the non-Hispanic Asian population, the sensitivity and positive predictive value dropped to 45% (95% CI 16%–75%) and 8% (95% CI 1%–14%), respectively. In the non-Hispanic Asian population, 92% (61/66) of pyrazinamide-monoresistant isolates were genotyped as *M. tuberculosis*.

## Discussion

In this large population-based epidemiologic study of *M. bovis* disease in California, we observed an increase in the annual percentage of TB cases attributable to *M. bovis* from 2003 through 2011. *M. bovis* disease accounted for nearly 25% of culture-positive TB cases in children. Patients with *M. bovis* disease were more likely than those with *M. tuberculosis* disease to die during treatment, and most deaths were among adults with concurrent immunosuppressive conditions. Hispanic ethnicity (compared with non-Hispanic white), extrapulmonary disease, and diabetes and other concurrent immunosuppressive conditions (excluding HIV co-infection) were independently associated with *M. bovis* disease compared with *M. tuberculosis* disease.

During 2010–2011, all (6/6) child case-patients with *M. bovis* disease had >1 parent/guardian who had been born in Mexico, and 4 of the 6 children had lived in Mexico for >2 months. These results, in conjunction with the a high percentage of extrapulmonary disease among the case-patients and the lack of *M. bovis* disease among children <12 months of age, are consistent with findings in previous epidemiologic studies that suggested that human *M. bovis* disease in the United States often results from consumption of unpasteurized dairy products originating from foreign countries, including Mexico (*6*,*12*,*22*,*23*). National pasteurization requirements, strict regulations on the importation of dairy cattle from Mexico into the United States, and an effective US bovine TB eradication program have substantially reduced *M. bovis* infection in US-born cattle (*22*,*24*). In the past century, the prevalence of *M. bovis* disease in US dairy herds has decreased from ≈5.0% to <0.001% (*25*). Furthermore, *M. bovis* isolates from humans in San Diego have been found to be genetically related to *M. bovis* strains from cattle in Mexico (*22*). Expansion of *M. bovis* disease surveillance and genotyping to include whole-genome sequencing may add discriminatory power beyond traditional genotyping and help to identify the source and route of transmission of *M. bovis* (*26*). Sharing of whole-genome sequencing data between countries and different health agencies may enhance future national and international prevention interventions.

Despite the relatively high percentage of extrapulmonary disease, 57% of *M. bovis* case-patients in our study had pulmonary involvement; this percentage is consistent with findings in other studies in the United States (*7*,*8*). Person-to-person transmission of *M. bovis* is considered infrequent, but the magnitude of such transmission has not been precisely quantified (*27*). Our findings are consistent with those from past research, which has shown that case-patients with *M. bovis* and *M. tuberculosis* pulmonary disease do not differ significantly in 2 of the key indicators of infectivity: presence of lung cavitation on chest radiographs and presence of acid-fast bacilli in sputum smears (*6*,*7*,*28*). In addition, previous research from pulmonary TB contact investigations showed that TB infection conversion rates among contacts did not differ significantly by mycobacterial species of the source case, suggesting that *M. bovis* is equally as transmissible as *M. tuberculosis* (*28*). However, current TB contact investigation guidelines do not include risk factors for *M. bovis* transmission. Although it is recommended to prioritize immunocompromised contacts of *M. tuberculosis* patients for evaluation, our data suggest that it may be even more important to prioritize immunocompromised contacts of *M. bovis* patients during contact investigations.

In our evaluation of the definition of pyrazinamide monoresistance, we found that isolates genotyped as *M. tuberculosis* may have been misclassified as *M. bovis* because of the pyrazinamide monoresistance definition. Misclassifications occurred most notably among the non-Hispanic Asian patients and might be explained by host, environmental (e.g., regional programmatic differences in TB treatment), and bacterial characteristics. From a recent national multivariate analysis of pyrazinamide resistance, Kurbatova et al. (*15*) suggested that bacterial lineage, not host characteristics, was the primary association between pyrazinamide monoresistance and *M. tuberculosis* disease.

Despite the possible definition-associated overestimation of *M. bovis* disease, the results from our study may still underestimate the true burden of *M. bovis* disease in California. Cases in children may be underestimated in the study population because sputum or gastric aspirate specimens are not consistently obtained from young children (*29*). In addition, *M. bovis* disease may be present in case-patients in the culture-negative subpopulation. We found case-patients with culture-negative TB to be similar to case-patients with culture-confirmed *M. bovis* disease with respect to age, extrapulmonary disease, and Hispanic ethnicity (data not shown).

Given the limitations of traditional genotyping and surveillance, we could not assess whether the increase in the number and percentage of adult *M. bovis* case-patients was to the result of recently acquired TB or reactivation of a previous infection. In addition, analysis of the new variables that were added to the national TB surveillance system in 2010 was hindered by the small number of child case-patients with *M. bovis* disease during 2010–2011. Also, because we did not conduct a medical chart review, we could not determine the cause of death among TB case-patients who died before the completion of TB treatment. Because of limitations in the national TB surveillance report form, we were unable to assess several possible relevant risk factors, including consumption of unpasteurized dairy products and the potential protective effect of the BCG vaccine against *M. bovis* disease. Although there is little information in the literature on the efficacy of the BCG vaccine in protecting against human *M. bovis* disease, the vaccine is notably protective against extrapulmonary disease and childhood TB disease, both of which are characteristic of *M. bovis* disease (*30*–*33*).

In summary, human *M. bovis* disease incidence has not declined in California, and the percentage of TB cases attributable to *M. bovis* has increased, exceeding the overall average for the United States. In California, there are ongoing interventions designed to limit the demand for and distribution of unpasteurized and contaminated dairy products, which are associated with *M. bovis* disease and other foodborne diseases (*23*). Elimination of human *M. bovis* disease in California likely requires further implementation of programs to reduce *M. bovis* contamination of dairy products in countries that have bovine TB, including pasteurization and test and cull interventions. In the interim, actions in the United States can help facilitate this effort. For example, *M. bovis* genotyping surveillance could be expanded and key risk elements (e.g., consumption of unpasteurized dairy products) could be captured in the national TB surveillance system to help determine *M. bovis* transmission routes. In addition, current TB contact investigation guidelines should be changed to include risk factors for *M. bovis* transmission. Last, future education efforts to prevent acquisition of *M. bovis* should focus on Hispanic binational families and adults with concurrent immunosuppressive conditions, including diabetes.
